# Educators' working conditions in a day care centre on ownership of a non-profit organization

**DOI:** 10.1186/1745-6673-6-36

**Published:** 2011-12-22

**Authors:** Bianca Kusma, Stefanie Mache, David Quarcoo, Albert Nienhaus, David A Groneberg

**Affiliations:** 1 Institute for Occupational, Social and Environmental Medicine, Theodor-Stern-Kai 7, 60590 Frankfurt am Main, Germany; 2 Department of Respiratory Medicine, Hannover Medical School, Carl-Neuberg-Straße 1, 30625 Hannover, Germany; 3 Department of Medicine/Psychosomatics, Charité - Universitätsmedizin Berlin, Free University and Humboldt University, Luisenstraße 13a, 10117 Berlin, Germany; 4 Institution for Statutory Accident Insurance in the Health and Welfare Services, Pappelallee 35/37, 22089 Hamburg, Germany

**Keywords:** educator, working conditions, task analysis, workload, real-time observation

## Abstract

**Background:**

Working conditions of nursery school teachers have not been scrutinized thoroughly in scientific research. Only a few studies have so far examined work-load and strain in this profession. Preferably, subjective perceptions should be corroborated by data that can be quantified more objectively and accurately. The aim of the present observational field study was to evaluate pedagogical staffs' workflow.

**Methods:**

In 2009 eleven educators in a day care centre were observed throughout three complete workdays. A total of 250 working hours were recorded.

**Results:**

An educators' workday lasted on average 07:46:59 h (SD = 01:01:10 h). Within this time span, an average of 02:20:46 h (30.14%, SD = 00:28:07 h) were spent on caring, 01:44:18 h on playing (22.33%, SD = 00:54:12 h), 00:49:37 h on educational work (10.62%, SD = 00:40:09), and only 00:05:38 h on individual child contact (1.21%, SD = 00:04:58 h).

**Conclusion:**

For the first time, educators' workflow in day care centres was studied in real time. Some of the educators' self-reported problems were corroborated. The results of this study form a basis upon which further investigations can be built and measures can be developed for an overall improvement of child care.

## Introduction

The PISA study (Programme for International Student Assessment) of the OECD comparing education among 15-year-olds in more than 30 countries showed that scholar performance of German pupils ranked low in the list of participating countries. The study also found that children who went to kindergarten or pre-school education achieve better results. Therefore more attention has been paid to day care centres as first socializing institutions [1]. Working conditions of pedagogical staff are not very well studied. Nevertheless this profession is subject to several psychosocial requirements [2]. Stress in this job is mainly caused by an interaction of minor strains which sum up in their negative effect [3]. Beside caring and educational duties pedagogical staff is confronted with additional tasks from a changed market situation (e.g. increased competition, certifications for quality control, independent management). Educators are often overtaxed by these tasks. As a consequence of these job conditions nursery school teachers are susceptible to develop complaints like backache, nervousness, headaches and stress or components of job burn-out and mental satiation [4]

Research questions on how these demands have an effect on the work ability and the health status of employees have also not been examined. Only capable, healthy and content personal is able to give a good care for children [5]. Therefore it is important not only to maintain the health of pedagogical staff in day care centres but also to promote it.

A general strike of German nursery school teachers in 2009 expressed their dissatisfaction with current working conditions. Educators complained about: shortage of staff [1], unfavourable respectively long working hours and difficulties in contact with parents [2]. Roughly 25.000 nursery school teachers struggled for better working conditions, improved health protection and higher salaries.

Nursery school teachers criticise in particular size of the group and an increased amount of paperwork. Research showed that a combination of both comes at the cost of direct child contact. In addition, educators have the feeling that they are not able to advance and support all children sufficiently [1,6,7].

A few questionnaire studies exist on work-load and strain in this profession but they are mainly based on self-reports. At present objective data is not available [1,8]. Nevertheless relying only on subjective statements of nursery school teachers might increase the risk of bias problems [9]. Keeping that fact in mind, an objective work analysis was conducted to collect precise time data of educators' work tasks. The overall aim of the monitoring was evaluate pedagogical staffs' workflow, identify sources of stress, and to provide an informative basis for the development of approaches for prevention.

## Subjects and Methods

### Setting and participating educators

Data of the BASE study (Bidirectional Assessment of Stress, job satisfaction and work ability of Educators in day care centres [10]) was collected from 10/09 to 12/09. Prior to the beginning of the monitoring a written request was sent to the management of a randomly selected day care centre on ownership of a non-profit organization in Berlin. After receiving departmental approval, educators were invited to participate in the study on a voluntary basis.

Inclusion criteria were as follows: (1) the participants have to hold a degree as early childhood educator or an equal value in training and (2) they have to work at least 6 hours (h) a workday. Of the 28 employed educators, 11 were female educators, who met the eligibility criteria, agreed to participate in the study.

### Data collection method

Pedagogical staffs' workflow was observed and registered in real time as described in detail in Mache (2010) [11]. A trained observer shadowed an educator recording each performed task with an Ultra Mobile PC (UMPC; designed software Samsung Q1; Samsung Electronics GmbH, Schwalbach, Germany) [12] and a specially designed software.

Thirteen task categories with 38 sub-categories were defined in order to describe the majority of job tasks educators carry out both sequentially and simultaneously during a typical work day (see Table 1). By using this program information could be gained about main and secondary activities and quantitative information about direct child contact. All activities (main and secondary) were recorded in units of time [12].

**Table 1:** Tasks performed by educators by category

Category	Description of activity
Documentation and administrative tasks	Writing observation forms, documentation assessment for schools, keep a diary of the progress of the children
Child care	Change nappies, help children to change clothes
Meals	Providing of food, feeding of smaller children
Afternoon nap	Supervision of the afternoon nap
Contact to parents	Welcoming, parents' evenings
Educational work	Singing, sportive activities, preparation und conducting of small experiments
Cleaning	Cleansing of rooms and toys, plants and animal husbandry
Continuing education/Supervision	Attendance at continuing education, supervision of work
Individual contact	Settle a dispute, console a child, welcoming of a child, individual support
Walking	Walking around between tasks (e.g. inside and outside of the day care centre, excursion)
Rest period: Break	Time of recovery (e.g. lunch)
Playing	Playing with children, surveillance of playing children
Internal Communication/meetings	Conversation with other educators or other staff, telephone calls

### Content validity

The process of developing the taxonomy started with a literature review and interviews with experienced educational specialists (Table 1). Subsequently an observation phase of two workdays was carried out to approve the content validity of the task categories, after which the taxonomy was modified. The final version of the task list was generated and then implemented to code tasks performed by participating pedagogical staff.

### Interobserver reliability

An interobserver reliability testing took place. Two trained observers recorded tasks of the same nursery school teacher simultaneously but independently over a period of 7 h. An interobserver agreement of 86% was achieved.

### Data collection procedure

Participants were monitored a complete work day by a trained observer. Data collection took place only on weekdays (Monday to Friday). Each of the 11 educators was accompanied throughout three different work shifts. During the observational period all working activities of the subjects were captured in real time.

To minimize the Hawthorne effect (possibility that educators change their performance in response to being observed) the observer stood at a distance of at least 3 m from the educator and was not allowed to talk to her/him.

### Data Analysis

All collected data were entered into a Microsoft Excel 2007^® ^spreadsheet for analysis. Descriptive statistics were calculated by using SPSS software package for social sciences, Version 18.0.

## Results

### Demographic characteristics of participants

All eleven participants were female with an average age of 36.3 years (SD = 7.4 years, range 25-47 years). The overwhelming majority of participants hold a degree as early childhood educator (90.9%), only one hold a university degree in education. On average, the pedagogical staff hat 12.2 years of work experience (SD = 10.3 years).

### Activities performed by educators

A total of 33 work days were monitored. This corresponds to 250 working hours. An average work day lasted 07:46:59 h (SD = 01:01:10 h). During this time a mean of 00:27:28 h (SD = 00:12:13 h) were reserved for breaks. The five most frequent performed tasks were: play (01:44:18 h; SD = 00:54:12 h), meals (01:14:27 h; 00:17:17 h), walking (01:13:41 h; SD = 00:37:23 h), educational work (00:49:37 h; SD = 00:40:09 h) and child care (00:40:55 h; SD = 00:13:25). The average times and percentages of each main work-related activity performed by educators are summarised in table 2.

**Table 2:** Average times for main activities performed by educators

	Sum main	Average	SD main	
Categories	activity	time main activity	activity	Percentage
	(hh:mm:ss)	(hh:mm:ss)	(hh:mm:ss)	
Documentation and administrative tasks	07:00:43	00:12:45	00:10:13	2.73
Child care	22:30:16	00:40:55	00:13:25	8.76
Meals	40:56:55	01:14:57	00:17:17	15.94
Afternoon nap	13:58:14	00:25:24	00:25:50	5.44
Contact to parents	03:39:24	00:06:39	00:10:15	1.42
Educational work	23:17:16	00:49:37	00:40:09	10.62
Cleaning	05:44:59	00:10:27	00:07:44	2.24
Continuing education/Supervision	00:15:01	00:00:27	00:01:29	0.10
Individual contact	03:05:54	00:05:38	00:04:58	1.21
Walking	38:31:39	01:13:41	00:37:23	15.78
Breaks	15:06:34	00:27:28	00:12:13	5.88
Playing	57:21:48	01:44:18	00:54:12	22.33
Communication	19:21:55	00:35:13	00:28:56	7.54

### Caring

The main tasks of a regular workday (02:20:46 h, SD = 00:28:07 h) can be assigned to the category "caring" (e.g. child care, meals and supervision of the afternoon nap). A nursery school teacher spent an average of 01:14:27 h (SD = 00:17:17 h) a day on preparing food and feeding smaller children. The mean daily duration of child care (e.g. changing nappies, helping children to change clothes) amounted to 00:40:55 h (SD = 00:13:25 h). Additionally 25 min (SD = 00:25:50 h) were dedicated to supervision of the afternoon nap.

### Playing

Another substantial block of time was allotted to the category "playing". During the study period nursery school teachers spent a total of 57:21:48 h on playing with children, which correspondents to 22.33% of all work activities. On average each educator spent 01:44:18 h (SD = 00:54:12 h) daily on this task.

### Educational duties

On the whole, ten per cent of the time recorded was spent on educational work (23:17:16 h). This correspondents to 00:49:37 h daily (SD = 00:40:09 h). Activities in this category mostly involved singing, dancing and other sportive activities as well as preparation and conducting small experiments.

### Internal communication and meetings

On average each nursery school teacher dedicated 00:35:13 h per day (SD = 00:28:56 h) to meetings and internal communication. In addition 01:34:53 h (SD = 00:35:14 h) were spent on internal communication and meetings as simultaneous tasks.

During the study period only one educator took part in continuing education (M = 00:00:27 h, SD = 00:01:29 h).

### Administrative task

Each educator spent average time of 00:12:45 h (SD = 00:10:13 h) on documentation duties. If simultaneously performed tasks were included an additional 00:07:15 h (SD = 00:03:55 h) per day were dedicated to administrative tasks. These activities principally comprised writing observation forms and took up an average of 00:06:48 h (SD = 00:07:28 h).

### Individual contact with children and communication with parents

During the study period educators spent a total of 03:05:54 h on individual contact to children. This corresponds to an average time of 00:05:38 h (SD = 00:04:58 h) per day. This task includes the sum of time allotted for settling of disputes consoling of a child as well as welcoming a child and individual support with educational tasks. Additionally 01:02:25 h (SD = 00:35:39 h) were shared between individual contact with children and other tasks.

It was observed that nursery school teachers spent 00:06:39 h (SD = 00:10:15 h) per day on communication with parents. These six min of communication included welcoming and parents' evenings.

### Additional tasks

The participating nursery school teachers spent an average of 01:13:41 h walking between tasks (SD = 00:37:23 h).

Another 00:10:27 h (SD = 00:07:44 h) were allotted for cleansing of rooms and toys as well as plants and animal husbandry.

### Multitasking

Educators spent a total of 118:14:58 h (47.14%) performing two or more activities at the same time during the study period. This corresponds to 03:35:00 h (SD = 00:50:36 h) on an average shift. Table 3 gives a summary of the different simultaneous activities performed by pedagogical staff. The most common task was "surveillance of playing children" while simultaneously "talking to another educator"

**Table 3:** Average times for simultaneous activities performed by educators

	Sum simultaneous	Average time	SD simultaneous	
Categories	activity	simultaneous activity	Activity	Percentage
	(hh:mm:ss)	(hh:mm:ss)	(hh:mm:ss)	
Documentation and administrative tasks	03:59:24	00:07:15	00:03:55	3.37
Child care	03:21:25	00:06:06	00:06:40	2.84
Meals	04:22:36	00:07:57	00:04:13	3.70
Afternoon nap	00:01:56	00:00:04	00:00:12	0.00
Contact to parents	04:06:57	00:07:29	00:05:53	3.48
Educational work	02:26:02	00:04:26	00:04:26	2.06
Cleaning	02:03:12	00:03:44	00:02:04	1.74
Continuing education/Supervision	00:01:34	00:00:03	00:00:09	0.02
Individual contact	34:19:59	01:02:25	00:35:39	29.03
Walking	01:13:17	00:02:13	00:02:40	1.03
Breaks	00:00:00	00:00:04	00:00:13	0.03
Playing	10:07:42	00:18:25	00:19:26	8.57
Internal Communication/meetings	52:10:54	01:34:53	00:35:14	44.13

### Changing activities

Changes in activities were measured to obtain additional information about educators work flow. On average participants performed 24 different tasks per working hour (SD = 8.74). The busiest hour of the work day was the first where 37 tasks were performed.

**Figure 1:**
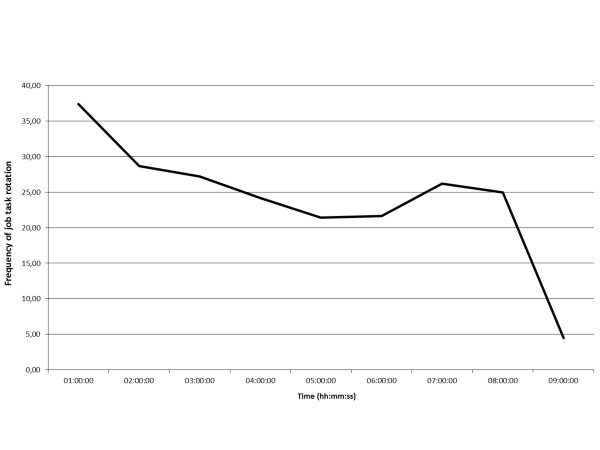
**Changes in activity during an average workday**.

The average frequency of job task rotations that educators do within single working hours are shown in Figure 1.

## Discussion

The current study analysed work activities of educators. To our knowledge, no computer-based real time studies do exist on working conditions in day care centres.

Our study revealed several important findings. Consistently with educators' perception relatively little time was allotted to individual contact to a child. This contact enables cognition of child's resources and abilities [13]. But a successful advancement of children requires enough time to build a relationship [14]. Therefore a reduction of the size of the group is needed.

A stable relationship between nursery school educator and child assists development, educational and learning processes [14]. This finding might be explained by the attachment theory. Close connections affect social behaviour and also self-perception, possibilities to interact as well as learning skills of a person. Attachment, education and literacy are a precondition, that a child can grow up in a holistic and positive manner [15]. However educator-child-relation is strongly affected by institution. Former studies showed that strain and workload have an influence on the relationships to children. Connections get formalised, which has negative consequences for children and educators [16].

Moreover, only a small amount of time was spent on contact to parents. Parental involvement is an important factor to mediate between educational institution and family structures. It is seen as a basic support of pedagogical work in the day care centre [17-19]. A successful exchange between nursery school teacher and parents could be beneficial for educational process of all relevant children. Former studies showed that family-supportive measures are particularly successful if parents and educators cooperate [20]. The use of manifold experiences of pedagogical staff and parents is meaningful for child's development. Furthermore a positive relationship between educator and parents is essential for a valuable child care. A parent should have the possibility to talk about any possible concern with the nursery school teacher, even more because educator are those - adjacent to physicians - who call parents' attention most frequently to developmental disorders of their children [21]. In this connection contact to parents is important with regard to preventive measures.

In opposition to nursery school teachers' reports in the current study only a small amount of time was devoted to documentation duties during study period. One reason therefore may be that educators have no time to fulfil these duties on the job. Research data confirm that pedagogical staff often completes these tasks during leisure time [22].

A source of stress is the high number of simultaneously performed tasks. One key result of the present study was the magnitude of multitasking in the workplace. As the principal reason for multitasking is reduction of time-pressure [23], this finding corroborates nursery school teachers' self-reports [1,6,24,25]. The demanding work environment compel nursery school teachers to perform two or more activities at the same time, although multitasking causes cognitive overload and has been found to be associated with reduced performance at work [26,27]. Moreover sequentially performed tasks last as long as simultaneously executed tasks [28]. Besides a reduced work performance multitasking may also affect the quality in child care.

## Limitations

Our study is subject to certain methodological limitations. First, our sample only consists of female nursery school teachers. Reason for this is the fact that the majority of educators are female in Germany. Therefore a focus should be set on male nursery school teachers in future investigations in order to assess gender differences in educators' work-loads.

Second, the number of participants monitored might be too small to be representative of all nursery school teachers. In the future, the present approach should be extended to a larger sample size. Additional studies are needed to replicate our findings in day care centres of different ownerships.

Third, the process of being observed might have influenced on the working behaviour of the educators (Hawthorne effect). Though it is too exhausting to adjust one' own work performance over a period of time. Therefore one can assume that observers' presence may not have a noteworthy effect on the general conclusions of the present investigation.

## Conclusion

The current study is the first of its kind to investigate the workflow of pedagogical staff in a German day care centre and as such, provides a valuable basis for future studies. The study results substantiate educators' statements about their working conditions with regard to size of the group, which comes at the cost of direct child contact. In addition the impact of time pressure was confirmed and which resulted in multitasking. Future studies should also investigate the rate of interruptions during work shift as many educators complain about it.

## Competing interests

The authors declare that they have no competing interests.

## Authors' contributions

BK and SM conceived and designed the study. BK managed the data assessment. BK analysed the data. BK wrote the manuscript. BK, SM, DQ, AN and DAG contributed substantially to its final version. All authors read and approved the final manuscript.
